# Complete Genome Sequence of *Atopobiaceae* Bacterium Strain P1, Isolated from Mouse Feces

**DOI:** 10.1128/MRA.00627-21

**Published:** 2021-07-15

**Authors:** Yasuha Watanabe, Nao Takeuchi, Jiayue Yang, Nozomu Obana, Kana Morinaga, Hiroyuki Kusada, Hideyuki Tamaki, Shinji Fukuda, Kazuharu Arakawa

**Affiliations:** a Japan Advanced Institute of Science and Technology, Nomi, Ishikawa, Japan; b Institute for Advanced Biosciences, Keio University, Tsuruoka, Yamagata, Japan; c Systems Biology Program, Graduate School of Media and Governance, Keio University, Fujisawa, Kanagawa, Japan; d Transborder Medical Research Center, University of Tsukuba, Tsukuba, Ibaraki, Japan; e Bioproduction Research Institute, National Institute of Advanced Industrial Science and Technology, Tsukuba, Ibaraki, Japan; f Faculty of Life and Environmental Sciences, University of Tsukuba, Tsukuba, Ibaraki, Japan; g Microbiology Research Center for Sustainability, University of Tsukuba, Tsukuba, Ibaraki, Japan; h Intestinal Microbiota Project, Kanagawa Institute of Industrial Science and Technology, Kawasaki, Kanagawa, Japan; i Faculty of Environment and Information Studies, Keio University, Fujisawa, Kanagawa, Japan; University of Rochester School of Medicine and Dentistry

## Abstract

*Atopobiaceae* bacterium strain P1 (*Actinobacteria*, *Coriobacteriales*) was isolated from mouse feces. Here, we report the complete genome sequence of this strain, which has a total size of 2,028,478 bp and a G+C content of 58.6%.

## ANNOUNCEMENT

Members of the order *Coriobacteriales* are Gram-positive, anaerobic bacteria and are well known to inhabit the mammalian intestinal tract. Some species of *Coriobacteriales* use mucin as a nutrient source (1, 2). It has been reported that diabetic patients have fewer mucin-utilizing bacteria, such as *Atopobium*, than do healthy subjects (3). Here, we report the complete genome sequence of *Atopobiaceae* bacterium strain P1, which was isolated from mouse feces, to elucidate the mucin degradation-related genes.

Mouse fecal samples were spread on Gifu anaerobic medium (GAM) agar (Nissui) supplemented with streptomycin (100 μg/ml), neomycin (100 μg/ml), and polymyxin B (50 μg/ml) (4) and were cultured at 37°C for 3 days under anaerobic conditions. Single colonies were transferred to GAM liquid medium and cultured at 37°C for 24 h under anaerobic conditions. The colony was identified by Sanger sequencing of the 16S rRNA gene. Genomic DNA was extracted using the Genomic-tip G/20 kit (Qiagen), and the long-read sequence library was prepared using a rapid barcoding kit (SQK-RBK004; Oxford Nanopore Technologies). The genomic DNA was not sheared or size selected before or during library preparation. Nanopore sequencing was performed with the FLO-MIN106 flow cell on a GridION device (Oxford Nanopore Technologies), and sequences were base called and adapter trimmed with GridION v19.10.2 software in high-accuracy mode. The quality was confirmed using NanoPlot v1.30.1. The Illumina sequence library used for error correction was prepared using the KAPA HyperPlus kit (Kapa Biosystems) and sequenced as 75-bp single-end reads with the high-output mode (75 cycles) of the NextSeq 500 sequencer (Illumina); base calling and adapter trimming were performed using bcl2fastq v2.18.0.12. The long-read sequencing produced a total of 552,256 reads (*N*_50_, 14.7 kbp). Assembly was performed using Canu v2.11.0 (5) with reads filtered for length over 39 kbp (411 Mbp in total, for estimated coverage of around 100×). The resulting single contig was circularized manually by deleting the overlapping ends. Unfiltered raw short-read sequences of 6.7 million reads were mapped to the assembly with Burrows-Wheeler Aligner (BWA) v0.7.11 (6). Subsequently, error correction was performed by three rounds of Pilon v1.23 polishing (7). The genome quality was evaluated with CheckM v1.1.3 (8), estimating genome completeness of 99.35% with the taxonomic family *Coriobacteriaceae*, and annotation of the genome sequence was performed using the DDBJ Fast Annotation and Submission Tool (DFAST) pipeline v1.1.6 (9). Default parameters were used to run the software unless otherwise specified.

The annotated genome of *Atopobiaceae* bacterium strain P1 has a total length of 2,028,478 bp with a G+C content of 58.6%, including 1,755 coding sequences, 52 tRNAs, and 6 rRNAs. Mucin degradation is a characteristic of this strain and is performed using a series of glucosaminidases. A similarity search for endo-β-*N*-acetylglucosaminidases (GH84) (10) using NCBI BLAST (11) revealed a highly similar protein, ATOBIA_N07120 (E value, <1e−20).

### Data availability.

The genome sequence reported here was deposited in DDBJ under accession number AP024608, and the raw reads were deposited in the Sequence Read Archive (SRA) under BioProject accession number PRJNA723828.
